# Melatonin and Indole-3-Acetic Acid Synergistically Regulate Plant Growth and Stress Resistance

**DOI:** 10.3390/cells11203250

**Published:** 2022-10-16

**Authors:** Min Zhang, Chunxue Gao, Ling Xu, Hui Niu, Qian Liu, Yixiao Huang, Guoshuai Lv, Hengshan Yang, Minhui Li

**Affiliations:** 1 Inner Mongolia Key Laboratory of Characteristic Geoherbs Resources Protection and Utilization, College of Pharmacy, BaoTou Medical College, Baotou 014040, China; 2 University Engineering Research Center of Chinese (Mongolia) Ecological Planting Medicinal Materials (Nurture) in Inner Mongolia Autonomous Region, College of agronomy, Inner Mongolia Minzu University, Tongliao 028000, China; 3 School of Life Sciences, Inner Mongolia University, Hohhot 010021, China; 4 College of Pharmacy, Inner Mongolia Medical University, Hohhot 010110, China; 5 Inner Mongolia Hospital of Traditional Chinese Medicine, Hohhot 010020, China; 6 Inner Mongolia Traditional Chinese & Mongolian Medical Research Institute, Hohhot 010010, China

**Keywords:** phytohormone, stress response, indole-3-acetic acid, melatonin, gene regulatory network, auxins

## Abstract

Plant growth and development exhibit plasticity, and plants can adapt to environmental changes and stress. Various phytohormones interact synergistically or antagonistically to regulate these responses. Melatonin and indole-3-acetic acid (IAA) are widespread across plant kingdom. Melatonin, an important member of the neuroendocrine immune regulatory network, can confer autoimmunity and protect against viral invasion. Melatonin functions as a plant growth regulator and biostimulant, with an important role in enhancing plant stress tolerance. IAA has a highly complex stress response mechanism, which participates in a series of stress induced physiological changes. This article reviews studies on the signaling pathways of melatonin and IAA, focusing on specific regulatory mechanisms. We discuss how these hormones coordinate plant growth and development and stress responses. Furthermore, the interactions between melatonin and IAA and their upstream and downstream transcriptional regulation are discussed from the perspective of modulating plant development and stress adaptation. The reviewed studies suggest that, at low concentrations, melatonin promotes IAA synthesis, whereas at high levels it reduces IAA levels. Similarly to IAA, melatonin promotes plant growth and development. IAA suppresses the melatonin induced inhibition of germination. IAA signaling plays an important role in plant growth and development, whereas melatonin signaling plays an important role in stress responses.

## 1. Introduction

During their growth and development, plants must adapt to various physiological responses triggered by environmental stress. These stress responses and the development of stress tolerance are driven essentially by plant hormones and their intricate crosstalk [1]. Plant hormones, small organic molecules with remarkable physiological effects even at very low concentrations, are universally involved in plant biological processes and regulate cell signal transduction to balance growth and stress responses [2]. Abscisic acid (ABA) is the most studied plant hormone involved in stress responses; however, other phytohormones such as auxin, cytokinin, melatonin, and brassinosteriod are also involved in plant responses to environmental stress. Synergistic or antagonistic interactions between different plant hormones can play a crucial role in multiple processes associated with plant responses to stress as well as other conditions.

Auxin, the first plant hormone discovered, affects multiple stages of plant growth and development. Auxins are generally considered to promote differentiation, as they initiate plant development and regulate the realization of organ morphology. However, the growth-promoting effects of auxins on plant stems, buds, and roots vary with concentration. Melatonin was first discovered in plants in 1995 and is now considered a regulator of plant growth. Similar to its observed effects in animals, melatonin has several specific functions in plant physiology, including regulating plant growth and conferring resistance to biological and abiotic stresses. 

Accumulating evidence from recent studies indicates that the interaction between indole-3-acetic acid (IAA) and melatonin plays an important role in plant growth and adaptation to stress responses. Genetic and biochemical studies have revealed the regulatory mechanisms of the IAA and melatonin signaling pathways. The identities of related genes have mainly been reported in *Arabidopsis thaliana*. At a certain low concentration, melatonin works in parallel with IAA and functions as its analog in promoting the induction, growth, and development of lateral and adventitious roots. At a high concentration, melatonin either reduces the IAA content, or maintains it at a constant level [3]. However, under semi-arid conditions, exogenous melatonin increases the content of endogenous IAA in maize, thus ensuring plant survival under stress and significantly improving maize yield [4]. However, the interaction between melatonin and IAA remains controversial, and the specific roles of these hormones have not been elucidated. Here, we review the research progress on crosstalk networks between melatonin and IAA, focusing on the upstream and downstream transcriptional regulation of key components between the two hormones. We also elucidate the signal exchange between melatonin and IAA which guides plant development and stress adaptation.

## 2. Biosynthesis of Melatonin and IAA

**Figure 1:**
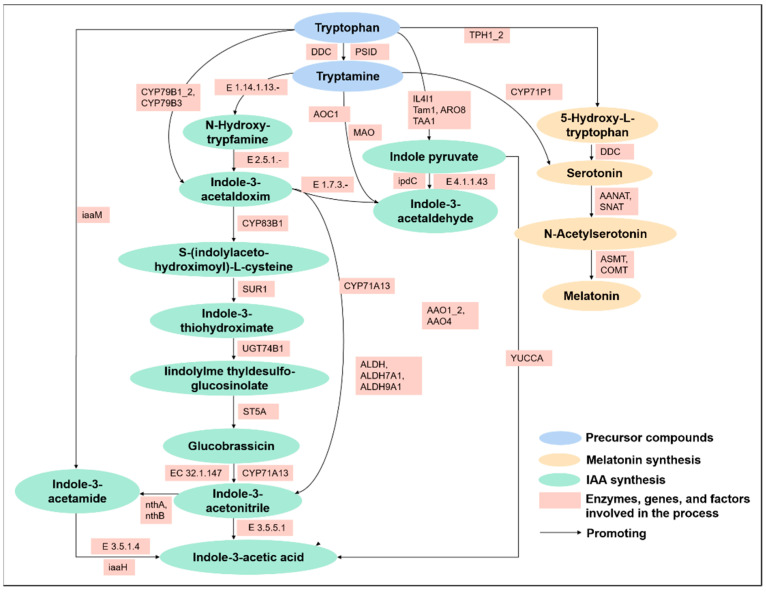
Synthesis pathways of IAA and melatonin in tryptophan metabolism.

At present, an increasing number of studies are investigating the metabolic pathways of melatonin and IAA. We found that tryptophan is the common precursor of both melatonin and IAA. We have summarized the synthesis of these hormones in the tryptophan metabolic pathway (Figure 1). Melatonin biosynthesis begins in a wide array of plant species through tryptophan. L-tryptophan decarboxylase (PSID) catalyzes the conversion of aromatic-L-amino-acid (DDC) into tryptamine, as shown in Figure 1; next, tryptamine 5-hydroxylase (CYP71P1) catalyzes tryptamine in serotonin, which is transformed by two steps to melatonin. Among some other species such as *Hypericum perforatum*, tryptophan, is catalyzed by tryptophan 5-hydroxylase (TPH) in 5-hydroxytryptophan, and then DDC transforms 5-hydroxytryptophan to serotonin. The same metabolic pathway is described for synthesis of melatonin in animals. The serotonin is converted to N-acetyl-serotonin in the next two steps, using serotonin N-acetyltransferase (SNAT)/arylalkylamine N-acetyltransferase (AANAT), and then catalysis of acetylserotonin O-methyltransferase (ASMT)/caffeic acid 3-O-methyltransferase (COMT) N-acetyl-serotonin results in melatonin. Tryptophan-is not only a provider of melatonin but also an indole-3-acetic precursor acid (IAA), perhaps implying that melatonin has a multifunctional role in plants. NADPH:oxygen oxidoreductase (N-hydroxylating) (E 1.14.1.13.-) catalyzes the conversion of tryptamine into N-hydroxytryptamine, which is then converted to indole-3-acetaldoxime under the action of N-hydroxyl-tryptamine oxidoreductase (E 2.5.1.-).

Tryptophan N-monooxygenase (CYP79B1_2)/CYP79B3 is used to directly transform tryptophan into indole-3-acetaldoxim. Next, aromatic aldoxime N-monooxygenase (CYP83B1) converts indole-3-acetaldoxim to S-(indolylaceto-hydroximoyl)-L-cysteine. S-(Indolylaceto-Hydroximoyl)-L-cysteine is converted to indole-3-thiohydroximate by S-alkyl-thiohydroximate lyase (SUR1), indole-3-thiohydroximate synthesizes lindolylme thyldesulfoglucosinolate through N-hydroxythioamide S-beta-glucosyltransferase (UGT74B1), Then, lindolylme thyldesulfoglucosinolate synthesizes glucobrassicin through aromatic desulfoglucosinolate sulfotransferase (ST5A), and glucobrassicin is synthesized into indole-3-acetonitrile catalyzed by myrosinase (E 3.2.1.147) and indoleacetaldoxime dehydratase (CYP71A13). Indole-3-acetaldoxim is directly transformed into indole-3-acetonitrile through CYP71A13 and aldehyde dehydrogenase (ALDH)/aldehyde dehydrogenase family 7 member A1 (ALDH7A1)/aldehyde dehydrogenase family 9 member A1 (ALDH9A1). Finally, indole-3-acetonitrile is transformed into indole-3-acetic acid (IAA) through nitrilase (E 3.5.5.1). 

Tryptophan is synthesized by tryptophan 2-monooxygenase (iaaM) into indole-3-acetamide, and indole-3-acetonitrile can also be converted into indole-3-acetamide by nitrile hydratase subunit alpha (nthA)/nitrile hydratase subunit beta (nthB). Amidase (E 3.5.1.4) and indoleacetamide hydrolase (iaaH) catalyze the conversion of indole-3-acetamide to IAA. Another IAA synthesis pathway is tryptophan catalyzed by L-amino-acid oxidase (IL4L1)/tryptophan aminotransferase (Tam1)/aromatic amino acid aminotransferase I (ARO8)/L-tryptophan---pyruvate aminotransferase (TAA1) to synthesize indole pyruvate, and then by indole-3-acetaldehyde oxidase (AAO1_2)/benzaldehyde dehydrogenase (AAO4) and indole-3-pyruvate monooxygenase (YUCCA) to synthesize IAA from indole pyruvate. It can be seen from the above that the upstream regulation of melatonin and IAA has been thoroughly studied at the present stage, and there is indeed a certain relationship between IAA and melatonin, which lays a foundation for our in-depth discussion on the signal transduction mechanism of melatonin and IAA.

## 3. Melatonin Signaling in Plants

Owing to the increasing achievement of interesting results in melatonin-related research in animals, researchers have turned their attention to the functions of melatonin in plants. Melatonin plays an important role in plant development and resistance to stress [5,6,7,8]. Its biosynthesis is induced by abiotic stressors such as cold, drought, and heavy metals, activating particular stress response factors. It participates in and regulates plant growth, promotes root growth after seed germination [9], affects the flowering time [10], and regulates sugar metabolism in plants [11]. The signal pathway of melatonin in plants has also been gradually elucidated. The first phytomelatonin receptor in *Arabidopsis thaliana*, candidate G-protein coupled receptor *2* (*CAND2*), a membrane protein that binds readily with melatonin, was identified in 2018 [12]. Treatment with exogenous melatonin at a 50 μM concentration upregulated the RNA polymerase genes *RPOTm* and *RPOTmp* by promoting the *CAND2* receptor and heterotrimeric G protein α subunit (*GPA1*) coupled to *CAND2* [13]. In *Arabidopsis*, *Cand2/pmrt1* is located on the plasma membrane (PM), interacts with *GPA1*, and regulates stomatal movement via the reactive oxygen species (ROS) signaling pathway mediated by *NADPH* oxidase [14].

**Figure 2:**
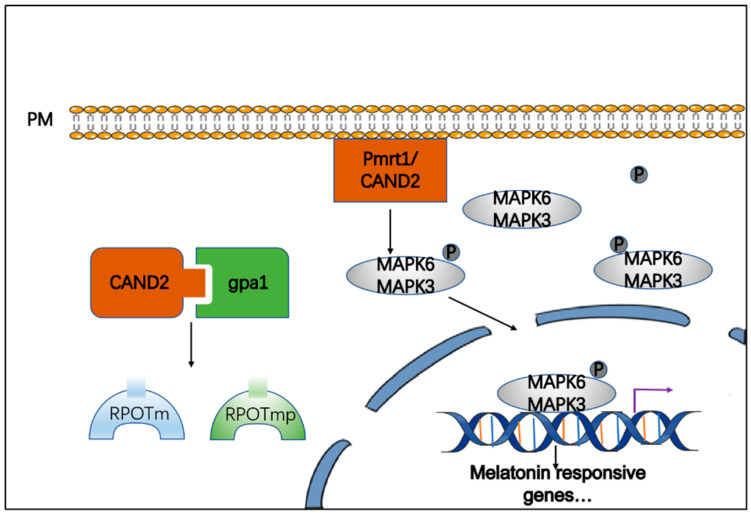
Melatonin signaling pathway from melatonin perception to action. Melatonin promotes the production of its receptor protein, enabling upstream MKK to activate MAPK3 and MAPK6, thereby activating melatonin signal.

Treatment with 1 μM melatonin activated MAPK3 and MAPK6 via upstream MAPK kinases (MKKs), including MAPKK4, MAPKK5, MAPKK7, and MAPKK9 [15]. However, this activation was not associated with G protein signaling, as the levels of G protein β did not change in a knockout mutant (*agb1*) of *Arabidopsis*. This indicated that melatonin induced innate immunity was triggered by MAPK signals from multiple MAPKK/MAPK components. MEKK activation is considered to be mediated via melatonin receptors or receptor kinases that recognize pathogen-derived molecular patterns (PAMPs) and effectors, leading to the activation of PAMP-triggered immunity (PTI) and effector-triggered immunity (ETI), respectively [16]. Melatonin can induce plant responses by activating *MAPKKK3* and oxidative signal-inducible 1 (*OXI1*) [17] (Figure 2).

Hydrogen peroxide (H_2_O_2_) and nitric oxide (NO) can regulate plant responses to environmental stress. The suppression of NADPH oxidase (RBOH) activity or the removal of H_2_O_2_ blocks the expression of stress-related genes, such as *CDPK1*, *MAPK1*, *ERF4*, and *ERD15*, further weakening melatonin induced defense responses [18]. NO and melatonin can interact to regulate glutathione (GSH) levels and glutathione reductase (GR) activity and maintain low levels of H_2_O_2_, thus regulating resistance to salt stress [19].

## 4. IAA Signaling in Plants

Auxin, which at low concentrations promotes plant growth, is critical for plant growth and stress resistance [20,21]. Its reported responses have become increasingly difficult to integrate into the accepted canonical auxin signaling pathways, and there is abundant literature on the noncanonical auxin signal transduction pathways. The auxin receptor transport inhibitor response 1 (TIR1) is central to both mechanistic pathways: its canonical pathway is localized in the nucleus and its noncanonical pathway in the cyto-plasmic matrix and plasma membrane [22]. The tir1-1 mutant of *Arabidopsis thaliana* shows defective root growth when heat shock protein 90 (*HSP90*) is inhibited. HSP90 forms a complex with the auxin receptor transport inhibitory response 1 (*TIR1*) and, when inhibited, impairs the nuclear localization of TIR1 and eliminates plant responses to auxins [23].

The IAA signal transduction pathway has been described in detail. Canonical auxin signal transduction mainly depends on the following three protein families: the F-box transport inhibitor response 1/auxin signaling F-box protein (*TIR1/AFB*) auxin co-receptors; the auxin/indole-3-acetic acid (*Aux/IAA*) transcriptional repressors; and the auxin response factor (*ARF*) transcription factors [24,25]. The accumulation of IAA leads to faster hydrolysis of the *Aux/IAA* protein and alleviates the inhibition of auxin response genes by allowing the formation of ARF dimers. Although this proposed model cannot explain the function of the ARF subtype, it is considered as an inhibitor. The *TIR1/ABF* protein binds to the *Aux/IAA* transcription inhibitor and participates in its polyubiquitination and subsequent proteasome-based degradation. This pathway rapidly induces auxin response genes—including *Aux/IAAs* and the auxin-responsive Gretchen Hagen3 (*GH3*) family of auxin homeostasis regulators—and subsequently triggers a negative feedback loop. However, when the IAA level is low, the *Aux/IAA* transcription inhibitors interact with ARFs and inhibit their activity [26]. 

The interaction between the *Aux/IAA* protein and the *SCF^TIR1^* complex is regulated by auxin, which simultaneously affects *TIR1* and related proteins. This pathway demonstrates the unique mode of action of SCF ligase; namely, auxin promotes the interaction between the *TIR1* and *Aux/IAA* proteins by binding to *TIR1* [27,28,29]. ARFs participate in a prominent noncanonical pathway. The N-terminal domains of ARFs also function as dimerization domains. Most ARFs have a conserved C-terminal Phox/Bem1p box (PB1) domain that is responsible for binding AUX/IAA proteins via the canonical auxin pathway. The ARF3 variant ETTIN (ETT) participates in noncanonical auxin ETT-signaling, regulating growth and tissue patterning in an IAA-dependent mechanism. 

**Figure 3:**
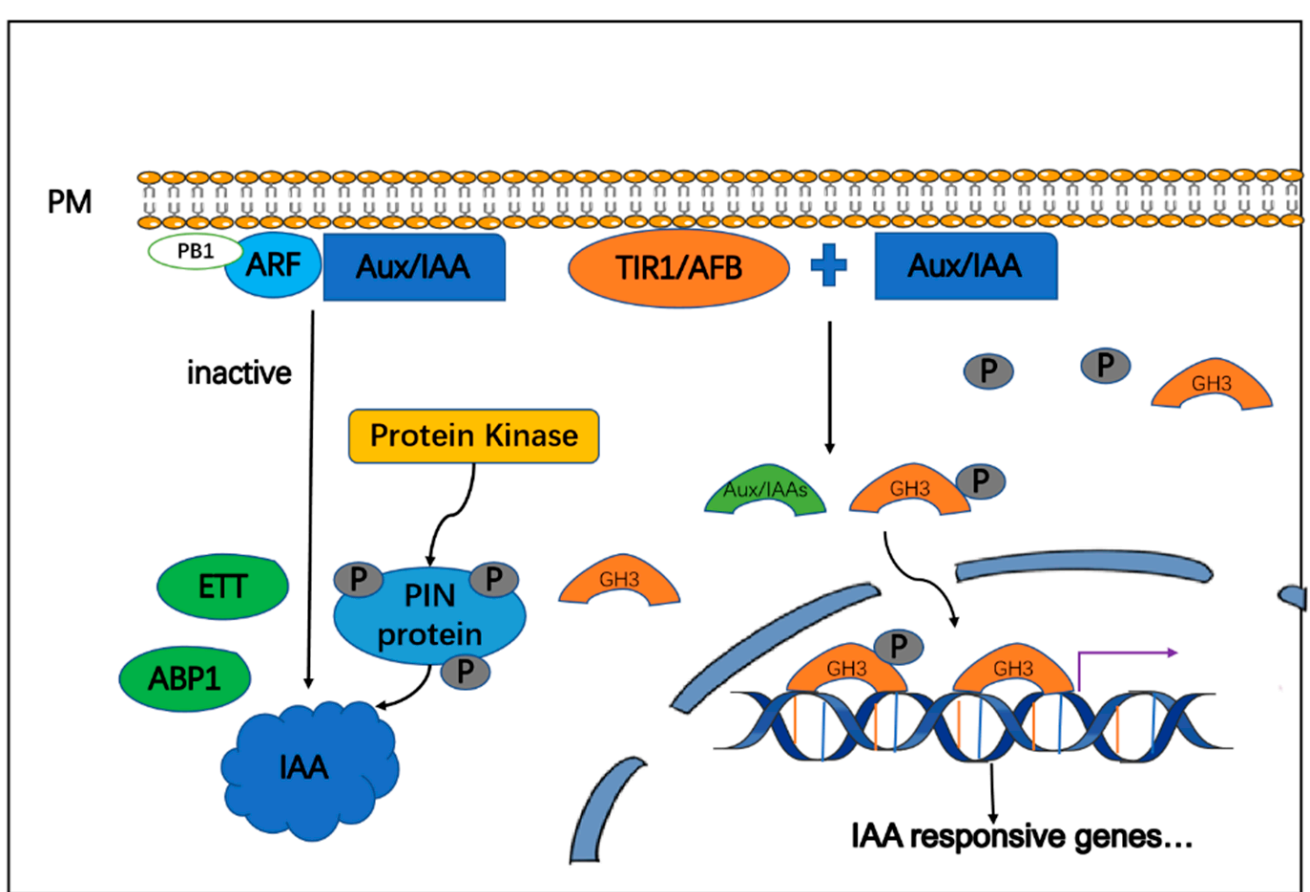
IAA signaling pathway from IAA perception to action. After receiving the IAA signal, the interaction between the three protein families of auxin and key components promotes the production of up-regulated gene encoding *GH3* protein to activate the downstream IAA signal.

Noncanonical auxin signaling is associated with various protein kinases, including D6 protein kinases, PINOID, mitogen-activated protein kinases [MAPKs], and PM-associated kinases. These contribute to the action of auxin by moderating PINFORMED (PIN) protein localization (phosphorylation) and activity [22]. It remains unclear whether auxin binding protein 1 (ABP1) functions as an auxin receptor. In Arabidopsis, ABP1 is secreted and binds specifically to the native auxin IAA at the typical acidic pH. These findings indicate that ABP1 is the auxin receptor for TMK1-based cell surface signaling, which mediates the global phospho-response and auxin canalization [30,31]. Both IAA and melatonin, therefore, play important roles in plant growth and resilience by interacting with MAPK kinases (Figure 3).

## 5. Relationships between the Melatonin and IAA Pathways

Melatonin and IAA have well documented synergistic effects on plant growth and development. Auxins and melatonin cooperatively induce adventitious root formation. The growth promoting activity of melatonin is one of its auxin-like effects. Treatment with exogenous melatonin has been shown to increase the content of auxin signal transduction genes (*IAA19* and *IAA24*) in tomato seedlings. NO acts as downstream signal of melatonin to enhance the auxin signal and induce adventitious roots in tomato explants [32]. Therefore, exogenous melatonin can activate the auxin signaling pathway. However, melatonin signaling also antagonizes IAA biosynthesis. In transgenic plants with melatonin overexpression, the decreased expression levels of key auxin biosynthesis genes (including *YUC1*, *YUC2*, *YUC5*, *YUC6*, and *TAR2*), as well as the effects of melatonin on auxin transport, significantly reduce the level of IAA in plant roots. These findings indicate that melatonin negatively regulates auxin biosynthesis [33].

IAA and melatonin signals can also be positively cross-regulated. Transcriptome analysis has revealed similar expression patterns of melatonin and auxin, confirming a partial overlap between the regulatory pathways of these hormones. The melatonin-mediated effects of the auxin pathway on root elongation were further investigated in plants that were auxin deletion mutants *yucQ* (*yc3*, *yuc5*, *yuc7*, *yuc8*, or *yuc9* mutations) or had been treated with inhibitors of auxin synthesis (L-AOPP) or auxin transport (TIBA). When auxin synthesis was completely inhibited, there was no obvious effect of melatonin on root elongation, suggesting that this effect is preconditioned on the presence of auxin [34]. Optimal concentrations of IAA (0–0.2 mg/L) and melatonin (0–0.2 mmol/L) in liquid MS medium promoted the growth of hairy roots in *Isatis indigotica*, whereas higher concentrations caused the de-differentiation of hairy roots. Treatment with 0.2 mg/L IAA and 0.2 mmoL/L melatonin increased the activities of enzymes (superoxide dismutase [SOD], peroxidase [POD], and catalase [CAT]) and the indirubin content of *Isatis tinctoria* hairy roots under salt stress, and melatonin had a greater effect than IAA [35]. In *Helianthus tuberosus* L., IAA improved the growth index to a greater extent, whereas melatonin had greater effects on photosynthesis and the antioxidant system. Moreover, melatonin promoted root elongation, dependent on the presence of IAA. These findings indicate that the combination of these enzymes can greatly improve stress resistance in plants [36].

### 5.1. IAA Signaling Regulation by Melatonin

Melatonin shares similarities with IAA in terms of its chemical structure and biosynthetic pathway, suggesting a potential link in signal transduction [37,38]. Melatonin treatment can increase IAA levels, and melatonin exhibits similar activity to IAA [39], with similar effects on plant growth and development. For example, melatonin promotes plant flowering, photosynthesis, senescence, and seed development [37,40,41]. These phytohormones co-regulate plant growth and development. When seeds were treated with melatonin and IAA simultaneously, IAA inhibited the inhibitory effect of melatonin on seed germination, indicating that IAA antagonizes melatonin in the regulation of seed germination [42]. Using the IAA-responsive marker construct *DR5::GUS,* it was demonstrated that the effects of melatonin on *Arabidopsis* roots are independent of IAA signal transduction [36]. However, other researchers have reported that melatonin has both positive and negative effects on the production of endogenous growth hormones. For example, in *Arabidopsis*, several IAA-related transcription factors (Including *NAC019*, *TCH4*, *FLA8*, and *PIN5*) were up- or down-regulated after treatment with melatonin [43]. In another root growth study on *Arabidopsis*, melatonin was shown to regulate IAA distribution by regulating IAA transport, thereby enhancing lateral root development. This indicated that melatonin and IAA synergistically promote lateral root development in wild-type *Arabidopsis thaliana* [44]. Of the 16 IAA-related genes identified, 12 genes (*AXR3*, *At1G29500*, *SAUR68*, *TT5*, *At4G38860*, *At4G00880*, *TT4*, *PIN5*, *At2G21050*, *CYP83A1*, *WAG1*, and *SHY2)* were found to be down-regulated by melatonin and 4 genes (*ACS8*, *At3G12830*, *AtGSTU1*, and *GH3.3)* were up-regulated, suggesting a potential role of melatonin in regulating IAA transport [44]. A comprehensive analysis of IAA synthesis, the *PIN* protein, and IAA reaction markers in *Arabidopsis* also showed that melatonin regulated the root meristem by inhibiting IAA synthesis and polar IAA transport [33].

Melatonin plays a protective role against oxidative stress, and oxidative stress can increase plant melatonin levels [45,46,47]. Melatonin promotes tolerance to low temperatures and osmotic stress, reduces physiological damage from stress, and improves salt tolerance and resistance to fungal diseases in plants [48,49]. Most studies on the roles of auxins in abiotic stress have focused on water-related (particularly drought), salt, and heavy metal stress [50]. However, IAA and melatonin also interact with each other to jointly promote plant resistance to biotic and abiotic stresses. A study identified 51 genes related to IAA response and signaling (including *MDC12*, *PBS3*, *ATGSTU1*, *HAI1*, *AT1G63840*, *ACS8*, *AT1G63720*, *TCH4*, *BT2*, *AT3G51660*, *CNI1, MPK11*, and *GH3.3*) that were altered by melatonin. Among these, 29 genes (including *MDC12*, *MPK11*, *GH3.3*, *ZF2*, *NAC019*, *PMZ*, and *SYP122*) were up-regulated and 23 genes (including *AT2G41820*, *PG2*, *Prx37*, *FLA8*, *AT1G31710, UGT74B1*, *WAG1*, *TT4*, *PIN5*, and *AUX1*) were down-regulated in response to melatonin. The study also found that most of the IAA-responsive genes down-regulated in response to melatonin were involved in IAA transport and homeostasis [43]. In addition, one of the up-regulated genes encodes GH3 protein, an IAA-amino synthase that combines amino acids with IAA to inactivate it [51]. This suggests that *Arabidopsis* seedlings respond to excess auxin in response to high levels of melatonin. However, none of the known genes on the IAA biosynthetic pathway (including *TAA*, *YUC*, *CYP79B*, *CYP450*, *TDC*, *AMI*, and *TIR*) showed significantly altered expression in response to melatonin [52].

The YUCCA (*YUC*) proteins, tryptophan aminotransferase of *Arabidopsis* (*TAA*) family, and *TAA*-related *1* and *2* proteins play important roles in the biosynthesis of IAA. Wang et al. [33] explored the effects of different concentrations of melatonin on the transcription levels of these genes. The transcription levels of *YUC1*, *YUC2*, *YUC5*, *YUC6*, and *TAR2* decreased significantly after treatment with 600 µM melatonin, whereas those of *YUC3*, *YUC4*, *YUC7*, and *YUC8* increased after treatment. The relative expression levels of *YUC3* and *YUC8* in roots were 1.5 times lower after treatment than those in the control. The *YUCCA* gene family contains key enzymes that catalyze IAA biosynthesis. A study analyzed the transcription levels of the *YUCCA* gene family after treatment with low concentrations of IAA and melatonin and found that both *YUCCA5* and *YUCCA8* were down-regulated. This indicated that the promoting of root length in wild-type *Arabidopsis* at low concentrations of melatonin was closely related to IAA [34]. In addition, the effects of melatonin treatment on *PIN* proteins (especially *PIN1*, *PIN3*, and *PIN7*) were also explored. The authors found that treatment with 600 µM melatonin significantly reduced the levels of *PIN1* in pre-culture and of *PIN3* and *PIN7* in the root cap and pre-culture area. This indicated that melatonin treatment inhibited the expression of *PIN1*, *PIN3*, and *PIN7*. Experiments on root development also showed that *PIN1/3/7* played important roles in the melatonin mediated inhibition of the root meristem.

The biosynthetic pathways of melatonin and IAA share the same substrate, tryptophan. Low concentrations of exogenous IAA increase melatonin production, whereas high concentrations of melatonin reduce IAA production and the levels of *PIN1/3/7* in *Arabidopsis* roots [44]. These dual actions of exogenous melatonin also lead to high levels of IAA in the cytoplasm and an increase in lateral root growth. At present, it is unclear how cytoplasmic IAA regulates lateral root growth. One possibility is that cytoplasmic IAA increases the level of cytosolic Ca^2+^ ions, and that increased calcium ion signaling ultimately leads to lateral root development [53]. Transcriptome analysis of the expression patterns of low concentration melatonin and IAA revealed many similarities between the two; for example, the genes regulated by melatonin and IAA were enriched in several common pathways in *Arabidopsis thaliana* [34]. The promotion of root growth by low concentrations of melatonin depends on the presence of IAA, and when the transport or synthesis of IAA is severely inhibited, melatonin cannot promote root growth. 

Studies on the interaction between melatonin and IAA have typically lacked a direct comparison between melatonin and IAA treatments under the same experimental conditions. In a recent study, *DR5::GFP* and *AOX1a::LUC* were used as markers for IAA response and mitochondrial retrograde signaling, respectively. These were used together with melatonin or IAA-treated Arabidopsis rosette leaves, and transcriptome analysis was conducted to investigate the potential molecular crosstalk between melatonin and IAA [54]. The results showed that melatonin treatment did not affect the expression of the IAA-responsive *DR5::GFP* gene in *Arabidopsis* seedlings. Unlike IAA, melatonin also did not affect mitochondrial retrograde signal transduction, but affected the expression of photosynthesis related genes. These results may indicate a trade-off between growth and defense. The small IAA up-regulating RNA (*SAUR*) family is one of three gene families known to be significantly elevated and transiently induced upon treatment with IAA [55]. The findings of several studies suggest that *SAURs* play an important role in IAA-mediated tissue root elongation in *Arabidopsis* and negatively affect IAA biosynthesis and polar IAA transport in rice [56]. IAA causes swelling of the cell wall [57], and melatonin may reduce the transcriptional level of genes in the *SAUR* family, thus reducing cell wall expansion and limiting the potential for pathogen invasion.

**Figure 4:**
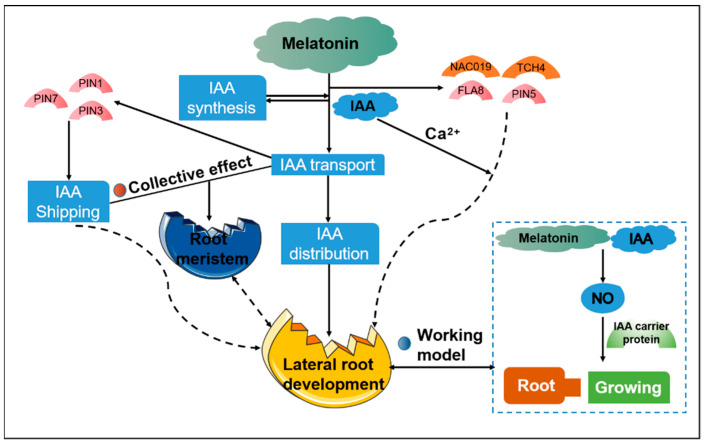
Regulation of IAA signaling by melatonin include key components and transcriptional regulation.

The aforementioned findings suggest that, in *Arabidopsis*, melatonin can regulate lateral and adventitious root induction in a parallel manner to IAA [58]. Melatonin can also interfere with IAA action by altering the IAA carrier, thereby altering the local IAA gradient [33,43,58]. Some researchers have proposed a working model to explain the roles of melatonin as an IAA-like regulator [59]. The signaling molecule NO is involved in various physiological processes during plant growth and development and is also an important regulator of stress responses and pathophysiological processes [60,61]. NO regulates plant roots, adventitious roots, lateral roots, root hair formation, and root geotropism [62,63,64,65]. The application of a NO donor can also mimic the effects of IAA. In *Arabidopsis thaliana*, auxin induces NO production in roots, mediated by nitrate reductase and the induction of S-nitrosothiols from proteins, regulating the activation of cell division and subsequent adventitious or lateral root formation, suggesting that NO plays a very important role in the induction of IAA [66]. Furthermore, NO mediates the IAA response, leading to the formation of dominant roots, and NO acts downstream of auxin [67]. One study reported that PINFORMED (*PIN*) proteins, specifically *PIN1*, *PIN3* and *PIN7*, are directly involved in auxin transport in plant roots, Melatonin treatment induced changes in apical IAA trafficking by up-regulating several PIN proteins (*PIN1*, *PIN3*, and *PIN7*) and IAA signaling genes (*IAA19* and *IAA24*). In contrast, the expression of these same proteins (*PIN1*, *PIN3*, and *PIN7*) is inhibited in *Arabidopsis*, thereby affecting root growth [33]. The proposed model suggests that the dual control of NO levels by melatonin and IAA may fine tune plant responses during growth, rooting, and tropicalization through the IAA carrier protein [48] (Figure 4).

### 5.2. IAA-Regulated Melatonin Signaling

In view of the similarity in chemical structure and biosynthetic pathway between IAA and melatonin, researchers have been exploring their effects on plant growth and development. A virus induced gene silencing (VIGS) technique was used to determine that inhibition of *DREB1α* and *IAA3* significantly reduced melatonin induced saline–alkali tolerance in *Solanum lycopersicum* (tomato). Furthermore, the physiological function of *DREB1α* and *IAA3* mediated melatonin in improving saline–alkali tolerance of tomato was further investigated. It was found that inhibiting *DREB1α* and *IAA3* significantly reduced the expression of genes related to ion transport, organic acid accumulation, stomatal movement, water retention and antioxidant enzymes in roots induced by melatonin. They found that *DREB1α* and *IAA3* are key downstream genes of melatonin induced saline–alkali tolerance in tomato, and the melatonin -*DREB1α*-*IAA3* cascade signaling network plays multiple roles in regulating tomato growth and stress tolerance balance [68]. The effects of exogenous 2,4-D (an auxin analogue) on endogenous hormones were detected in embryonic callus of *Eriobotrya prinoides var dadunens*. The results showed changes in melatonin related to culture time, first decreasing and then increasing after 30 days [69]. In *Arabidopsis thaliana*, the addition of auxin polar transport inhibitor *TIBA* indicated that the regulation of primary root growth by melatonin and cytokinin was dependent on auxin transport. This indicates that the polar transport of auxin plays an important role in this process [70]. However, the results indicate that the expected molecular regulatory mechanisms of auxin and melatonin are less well studied. This is a very promising area of future exploration. In addition, only a few genes are involved in their temporal regulation, which hinders research on their participation in plant stress. Gene expression analysis of auxin and melatonin regulation in response to various abiotic stresses is expected to elucidate the regulatory pathways of these hormones and the underlying molecular mechanisms.

### 5.3. Interactions between Melatonin and IAA Levels to Balance Growth and Adaptation to Stress

Melatonin, an important stress response hormone in plants, accumulates under adverse conditions such as drought, cold, flood, and heavy metal stress. This inhibits the production of auxin, as both auxin and melatonin are synthesized with tryptophan as the starting material. When IAA is the dominant signal in *Arabidopsis thaliana*, it is inhibited by the zinc finger protein (*ZAT6*); in contrast, *ZAT6* knockout plants are not sensitive to melatonin regulated auxin biosynthesis [71]. Most studies on the interaction between melatonin and auxin focus on plant roots. Although some studies have reported that melatonin induced changes in plant root development may not be related to auxin signaling [39], studies on rice have shown that melatonin directly or indirectly activates the auxin signaling pathway to shape root structure [72]. In addition, the promoting effect of melatonin on lateral root development disappeared in knockout mutants of auxin transport (including *pin5*, *wag1*, *tt4*, and *tt5*) in *Arabidopsis thaliana*. This indicated that melatonin and auxin synergistically promoted lateral root development in wild-type *Arabidopsis thaliana* [44].

IAA plays an important role in mediating root changes during abiotic stress [73]. The production of local maximum IAA inhibits cell elongation locally, thus preventing the emergence of lateral roots. In *Arabidopsis thaliana*, the local minimum IAA was found to trigger a transition from cell division to cell differentiation [74]. In the typical IAA signaling pathway, the binding of IAA to its receptor activates the E3 ubiquitin ligase complex SCF, resulting in the degradation of the *Aux/IAA* transcriptional repressor. This degradation allows the *ARF* to regulate the expression of IAA response genes [75,76]. In *Arabidopsis*, typical IAA perception includes six receptors that lead to the activation of target genes *TIR1* and five *AFB* proteins (*AFB1*–*AFB5*) [77]. The accumulation and differential perception of IAA in roots modulates several types of abiotic stresses [78,79,80]. A study on salt stress showed that different treatment times lead to responses from different genes. For example, *TIR1* was up-regulated after six hours of salt stress treatment [78], whereas *TIR1* and *AFB2* were down-regulated at the protein level after four hours of salt stress treatment [81]. Interestingly, the overexpression of *TIR1* (an anti-degradation form) endowed plants with a salt tolerant phenotype and increased the seed germination rate, lateral root density, and Na^+^ exclusion, among other parameters [78]. *AFB3* has been identified to play a key role under limited nitrate conditions, and its downstream signal components have also been identified [82]. For example, the *NAC4* transcription factor has been described as a signal component downstream of *AFB3* in response to nitrate [83]. Recent studies also show that *SZF1* is a key transcription factor in the IAA-dependent response to salt stress, as it regulates *NAC4* [84]. A study on drought stress revealed that, in *Arabidopsis*, some drought stress genes are regulated by IAA. Osmotic stress inhibits cell division during the growth of *Arabidopsis* leaves by inducing plant *ARFs*, at least in part [85]. In addition, other related genes have been found to play important roles in the abiotic stress response of *Arabidopsis*. Drought and salt stress significantly reduced the expression of *TSB1*, resulting in decreased levels of tryptophan and IAA [86]. The same study also reported that a large amount of H_2_O_2_ produced by plants under drought or salt stress could sulfenylate the cysteine at position 308 of the *TSB1* protein. This inhibited the tryptophan synthase activity of *TSB1*, thereby reducing the levels of tryptophan and IAA.

High temperature stress is known to have an important impact on the growth, development, and geographical distribution of plants. In *Arabidopsis*, high temperature stress can significantly induce the expression of a key transcription factor (*PIF4*) and an IAA synthesis-related gene (*YUC8*) in the light signaling pathway. In a study on heavy metal stress, researchers reported that aluminum stress can induce the specific ectopic expression of key IAA synthesis-related genes (*TAA1* and *YUCCA*) in the root tip transformation region. This caused the excessive accumulation of IAA in this region, thus inhibiting the elongation of main roots. This indicates that aluminum toxicity affects the normal growth of plant roots by regulating local IAA synthesis, polar transport, and corresponding signal transduction [87]. In addition, cadmium toxicity can also affect the growth and development of plants by disturbing the internal homeostasis of IAA [88]. Auxins can directly and positively regulate the resistance of plants to cadmium toxicity. This regulation is mainly achieved by reducing the transport efficiency of Cd^2+^ from root to aboveground parts and inducing the activities of some antioxidant enzymes, such as SOD, CAT, and POD. These findings indicate that some key genes related to auxin synthesis, metabolism, polar transport, and signal transduction are directly regulated by stress. However, the regulation mechanisms of auxin at the molecular level under conditions of abiotic stress need to be further examined in future studies. In addition, the molecular mechanisms of synergistic or antagonistic interactions between phytohormones under stressful conditions should also be investigated.

**Figure 5:**
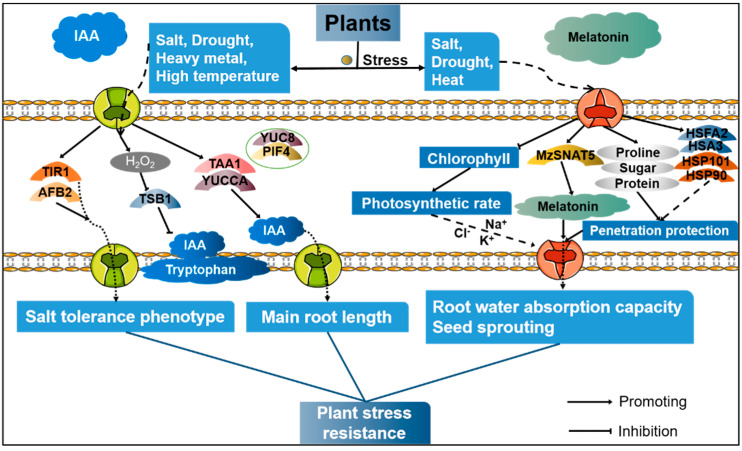
Regulation of melatonin signaling by IAA include key components and transcriptional regulation.

Melatonin has been shown to play an important role in plant responses to abiotic stress. A study of drought stress revealed that melatonin conferred resistance to oxidation, regulated infiltration, and promoted plant growth, thus slowing down the negative effects of drought stress on plants and helping plants adapt to the drought environment. Treatment with exogenous melatonin also increases the levels of proline, soluble sugar, and soluble protein in plants [89] and plays a role in the osmotic protection of cells [90], thus effectively improving the root water absorption capacity of seedlings under drought stress. In *Arabidopsis*, the ectopic expression of *MzSNAT5* can improve melatonin synthesis in the mitochondria, reduce oxidative damage, and improve the drought tolerance of plants [91]. Under salt stress treatment, melatonin can alleviate the damage caused by salt stress. The main physiological mechanisms of this include the following: inhibiting chlorophyll decomposition and increasing the photosynthetic rate [92]; enhancing the activities of antioxidant enzymes and reducing the ROS content [93]; and regulating the absorption and transport of Na^+^, Cl^−^, and K^+^ ions and regulating ion homeostasis [94]. Heat stress can cause changes in a series of metabolic processes, leading to the excessive production of ROS, photoinhibition, protein denaturation, damage to biofilm structure and function, and inhibition of protein synthesis [95]. However, melatonin significantly up-regulates the expression of heat shock factor *HSFA1* to activate several heat response genes—including *HSFA2*, HSA32, *HSP90*, and *HSP101*—thus improving the heat tolerance of *Arabidopsis* plants [96]. In addition, treatment with exogenous melatonin promotes the activity of antioxidant enzymes and improves the germination of *Arabidopsis* seeds under heat stress [97]. Endogenous melatonin also alleviates the toxicity of heavy metals, and the application of exogenous melatonin effectively alleviates heavy metal stress in plants. Taken together, these results indicate that plants can increase the production of endogenous melatonin in response to adverse environments, and that melatonin plays a crucial role in regulating a series of physiological processes (such as plant responses to abiotic stress) [98] (Figure 5).

When environmental stress conditions are alleviated and appropriate levels of water and temperature are provided, the endogenous melatonin level decreases, whereas IAA synthesis increases. The plant shifts from a poorly defended state and resumes growth. The interaction between the two hormone signals helps regulate the balance of key enzymes and substances, thus modulating plant growth and development and the transitions related to stress response.

### 5.4. Crosstalk between Melatonin and IAA in Other Plant Species

Melatonin and auxin have been shown to regulate growth and stress responses not only in Arabidopsis, but also in other plant species. Plant resistance to various stresses is associated with improved antioxidant capacity. In *Zoysia japonica*, melatonin not only increases the antioxidant capacity, but also affects the expression of IAA related genes, which play an important role in seed germination [99]. The combined use of melatonin and IAA can increase the levels of chlorophyll, total soluble protein, total soluble sugar, and ascorbic acid in wheat under stress, and can also enhance the antioxidant capacity of the plants [100]. Maize yield is limited in arid areas, and the application of exogenous melatonin can improve plant tolerance, thereby increasing maize yield. Corn seeds soaked with melatonin exhibit increased levels of zeatin + zeatin riboside, IAA, and gibberellic acid, which promote plant growth [4]. Both melatonin and IAA enhance plant tolerance to salt stress [101].

IAA and melatonin are important indole compounds in plants. Arnao et al. studied the growth of lupin plants [102] and various monocots [103] to examine these two indole compounds and reported their similarities. Since then, several other studies have demonstrated that melatonin can induce vegetative growth in a similar way to IAA, but through other means [59]. For instance, growth depends on melatonin concentration and tissue type. Therefore, roots are more sensitive to growth than aerial tissues (such as the talus and leaves). Growth is inhibited at high concentrations of melatonin, similar to IAA. Recent studies have also shown that low concentrations of IAA activate melatonin biosynthesis, whereas high concentrations of melatonin inhibit IAA biosynthesis. However, more studies have shown that melatonin regulates signaling elements such as *TIR1*, *Aux/IAA*, and *SAURs* to mediate the activation of growth processes [104].

Melatonin treatment has been shown to induce a slight (1.4–2.0-fold) increase in endogenous IAA, compared to untreated *Brassica napus* [105] and tomato [32] plants. However, in transgenic melatonin overproducing plants, IAA levels have been reported to drop significantly. Tomato plants overexpressing sheep *SNA HIOMT* and *Arabidopsis* plants overexpressing apple *HIOMT* had endogenous melatonin levels up to 6-fold higher than those of wild-type plants, whereas endogenous IAA levels decreased 7-fold in tomato and 1.4-fold in *Arabidopsis*. The phenotypes of these transgenic plants clearly resembled those of an auxin-like response, including the promotion of root growth and rooting, reduced apical dominance, and the induction of root primordia by melatonin independent of the IAA signaling pathway. IAA, but not melatonin, activated the auxin-inducible gene expression marker *DR5::GUS* in *Arabidopsis* [39,106]. These data suggest that melatonin can act in parallel with IAA in the induction of lateral and adventitious roots [103]. In cucumber, melatonin has been shown to up- or down-regulate approximately 320 genes related to root development. Some of these transcription factors and ethylene transcription factors can negatively regulate root-related genes, thus inhibiting root formation. Liang et al. found that IAA-related genes were significantly activated under melatonin treatment. Several transcription factors and candidate cis-regulatory elements involved in root growth and development and auxin-related processes are encoded by co-differentially expressed genes. This suggests that melatonin-mediated root growth occurs in an auxin signaling pathway-dependent manner. In addition, gravity response analysis has been used to identify the process by which melatonin affects auxin regulation in rice roots [72]. Melatonin regulates IAA distribution by regulating auxin transport, thereby promoting lateral root development [107]. Chen et al. found that the application of 0.1 mM melatonin promoted root growth, whereas 100 mM melatonin inhibited root growth. Furthermore, this stimulatory effect was only detected in 2-day-old seedlings, and older seedlings (4-day-old) appeared to be less sensitive to both the stimulating and inhibitory effects of melatonin. Exogenous melatonin (0.1 mM) also increased the levels of endogenous free IAA in roots, whereas higher concentrations of melatonin had no significant effect on endogenous IAA levels [105]. As a signal molecule, melatonin can regulate leaf senescence. Endogenous melatonin is also associated with the overexpression of *CsASMT* (a key gene for melatonin synthesis), which can delay the senescence of dark-stimulated leaves. A greenhouse study on cucumbers reported that melatonin inhibited leaf senescence by reducing ABA biosynthesis and inhibiting its signaling pathway, while promoting IAA biosynthesis and its signaling pathway [108]. In apple, melatonin promotes the formation of adventitious roots, mainly by promoting IAA levels and up-regulating *MdWOX11* in the stress induction stage [109].

## 6. Conclusions and Perspective

IAA is involved in almost all aspects of plant growth and development, including responses to external stimuli (such as biotic and abiotic stresses). Melatonin also plays an important role in plant growth and stress response. This paper summarizes the known literature on interactions between melatonin and IAA in regulating plant growth and development. First, melatonin regulates IAA levels and promotes IAA biosynthesis at low concentrations. Specifically, melatonin enhances core signal transduction genes related to IAA (such as *IAA19* and *IAA24*), several PIN proteins (*PIN1*, *PIN3*, and *PIN7*) and key genes related to IAA biosynthesis (*YUC1*, *YUC2*, *YUC5*, *YUC6*, and *TAR2*), thereby enhancing lateral root development. However, melatonin reduces IAA levels at high concentrations. The exogenous application of low concentrations of IAA also increases melatonin production. Second, melatonin has an IAA-like effect on plant growth and development, and can promote plant flowering, photosynthesis, senescence, and seed development. IAA can also modulate the antagonistic effects of melatonin on seed germination. When seeds are treated with both melatonin and IAA, IAA suppresses the inhibitory effects of melatonin on seed germination. Third, NO signaling molecules are involved in various physiological processes during plant growth and development and are also important regulators of stress response and pathophysiological processes. The dual control of NO levels by melatonin and IAA may regulate plant growth and environmental response through IAA carrier proteins. Finally, the combination of melatonin and IAA can improve the stress resistance of plants.

Both melatonin and IAA can regulate each other’s levels; however, the mechanisms of how plants regulate melatonin from the perspective of IAA need further exploration. In the future, systems biology approaches—especially combinations of different “omics” approaches with CRISPR/Cas9 technology—should be used to accelerate the identification of key genes related to the biosynthesis, degradation, and signaling pathways of melatonin and IAA. Analyzing the changes in melatonin content and signaling in mutant lines for IAA biosynthesis, degradation, and signal transduction will also help reveal the relationship between melatonin and IAA. Both of these hormones are known to communicate extensively with other hormone signaling pathways by regulating their biosynthesis or signal transduction. Therefore, uncovering the crosstalk between IAA and melatonin would contribute to our understanding of more complex networks of crosstalk among plant hormones.

## Data Availability

Not applicable.

## Abbreviations

Indole-3-acetic acid (IAA), indole-3-pyruvate monooxygenase (YUCCA), abscisic acid (ABA), reactive oxygen species (ROS), nitric oxide (NO), hydrogen peroxide (H2O2), transport inhibitory response 1 (TIR1), auxin (Aux), superoxide dismutase (SOD), peroxidase (POD), catalase (CAT).
